# Navigating the Current Landscape of Non-Clear Cell Renal Cell Carcinoma: A Review of the Literature

**DOI:** 10.3390/curroncol30010070

**Published:** 2023-01-09

**Authors:** Alexius John, Lavinia Spain, Anis A. Hamid

**Affiliations:** 1 Department of Medical Oncology, Eastern Health, Melbourne, VIC 3128, Australia; 2 Department of Medical Oncology, Peter MacCallum Cancer Centre, Melbourne, VIC 3000, Australia; 3 Eastern Health Clinical School, Monash University, Melbourne, VIC 3128, Australia; 4 Department of Surgery, University of Melbourne, Melbourne, VIC 3010, Australia

**Keywords:** non-clear cell, renal cell carcinoma, papillary, hereditary leiomyomatosis and renal cell cancer, chromophobe, medullary, collecting duct

## Abstract

Non-clear cell renal cell carcinoma (nccRCC) is an entity comprised of a heterogeneous constellation of RCC subtypes. Genomic profiling has broadened our understanding of molecular pathogenic mechanisms unique to individual nccRCC subtypes. To date, clinical trials evaluating the use of immunotherapies and targeted therapies have predominantly been conducted in patients with clear cell histology. A comprehensive review of the literature has been undertaken in order to describe molecular pathogenic mechanisms pertaining to each nccRCC subtype, and concisely summarise findings from therapeutic trials conducted in the nccRCC space.

## 1. Introduction

Renal cell carcinoma (RCC) is the seventh-most common malignancy globally [1], representing 5% of adult malignancies in males and 3% in females [2]. Recognised risk factors include cigarette smoking, obesity and hypertension. Underlying disorders such as von Hippel-Lindau (VHL) syndrome, end stage renal failure, acquired renal cystic disease, dialysis dependence and prior renal transplant predispose to development of RCC [3]. Clear cell RCC (ccRCC) constitutes approximately 80% of all RCC, whilst the less common non-clear cell RCC (nccRCC) can be further stratified by subtype [4]. The World Health Organisation (WHO) has updated the classification system of RCC in 2022, distinguishing subtypes on the basis of both morphological appearance as well as underlying molecular aberrations (e.g., TFE3 and ALK-rearranged RCC, SMARCB1-deficient medullary-like RCC), thus offering an integrated approach to characterizing tumour subtypes [5]. 

The majority of advanced RCC clinical trials are targeted to patients with ccRCC. Management paradigms within this space have historically consisted of cytokine therapy (such as interferon-alpha and IL-2) and oral targeted therapies (tyrosine kinase inhibitors, such as sunitinib); however, immunotherapy (IO) has recently emerged as a mainstay of initial treatment of metastatic ccRCC [6]. This shift has occurred due to improvements in survival with immunotherapy in large randomized clinical trials (CheckMate-214, KEYNOTE-426, CheckMate-9ER, CLEAR and JAVELIN-101) [7,8,9,10,11]. Notably, only patients with a clear cell component of RCC were included in these practice-informing trials. Comparatively, nccRCC- a heterogeneous group of malignancies with diverse clinical and molecular features- has been less well studied. Much of the treatment evidence for advanced nccRCC arises from smaller, non-randomised or retrospective studies and expert-level evidence only. Moreover, there remains a dearth of established treatments and clinical trials specifically designed for this patient population. Herein, we provide an overview of nccRCC with a focus on evidence for systemic therapy of advanced disease. 

## 2. Pathological, Molecular and Clinical Features of nccRCC Subtypes

Certain subtypes of metastatic nccRCC are associated with more aggressive disease behaviour and poorer survival outcomes compared to ccRCC. Within nccRCC, subtypes exhibit different clinical courses. The incidence of morphological subtypes of nccRCC are listed in Table 1. Recent genomic sequencing studies of nccRCC have revealed molecular aberrations amongst different nccRCC subtypes that parallel different patterns of clinical behavior, therapy response and prognosis. Herein, we discuss nccRCC subtypes with unique histological and molecular features in further detail.

### 2.1. Papillary

Papillary RCC is denoted histologically by the presence of basophilic cells arranged in a papillary fashion [13]. Papillary RCC can be further divided into two types, each demonstrating a unique biological basis and clinical phenotype. Type 1 papillary RCC is typically characterized by papillae with shallower layers of columnar epithelial cells that possess minimal cytoplasm and small ovoid nuclei. Additional features include presence of foamy macrophages and psammoma bodies [13]. In contrast, type 2 papillary RCC exhibits more aggressive histological features such as pleomorphic irregular nuclei with nucleoli and eosinophilic cytoplasm-containing large cells [14]. Tumour stage and grade at time of diagnosis are consequently higher with type 2 papillary RCC [13]. This translates into the clinical setting, with type 1 papillary RCC conferring a better prognosis than ccRCC and type 2 papillary RCC. It is known to have a more indolent clinical course and is less likely to metastasise than type 2 papillary RCC [14].

*MET*-activating mutations were initially found in the hereditary papillary RCC setting [15]. The pathogenicity of these mutations was subsequently demonstrated in both hereditary and sporadic forms of the disease in further studies reported in the Cancer Genome Atlas (TCGA) profiling [16]. A total of 81% of type 1 papillary RCC demonstrates alteration of *MET* or increased copy number of chromosome 7, the long arm of which contains the MET proto-oncogene [16]. In contrast, type 2 papillary RCC has a more heterogeneous molecular profile. Recurrent genomic alterations described in this subtype include *CDKN2A* mutation/promoter hypermethylation, *NRF2-ARE* overexpression, *TFE3* fusions and *SETD2* mutations [16]. 

### 2.2. Hereditary Leiomyomatosis and Renal Cell Cancer (HLRCC) 

HLRCC is a rare subset of nccRCC with an autosomal dominant pattern of inheritance that predisposes to the development of cutaneous and uterine leiomyomas in addition to RCC [17]. Historically, HLRCC was classified as type 2 papillary RCC. It is now recognised as a distinct entity [5], as it is pathogenically driven by germline mutations in the fumarate hydratase (FH) gene. FH is responsible for the formation of L-malate from fumarate as part of the tricarboxylic acid (TCA) cycle. Mutations in FH lead to enhancement of hypoxia-inducible factor 1-alpha (HIF-1α) and downstream factors such as erythropoietin (EPO) and vascular endothelial growth factor (VEGF), in turn leading to tumorigenesis [18]. 

### 2.3. Chromophobe

Chromophobe RCC originates from the intercalated cells of the renal collecting duct [19]. Loss of whole chromosomes is a hallmark of chromophobe RCC. A higher frequency of mutations in the tumour suppressor genes TP53 and PTEN have been shown in this subset of nccRCC. TERT rearrangements may also play a role in tumorigenesis of chromophobe RCC [20]. Genetic syndromes associated with chromophobe RCC include the following: tuberous sclerosis, Cowden syndrome, Birt-Hogg-Dube syndrome, hereditary paraganglioma/phaeochromocytoma and BAP1 tumour predisposition syndromes [21]. Clinically, chromophobe RCC is more likely to arise in young female patients and has an indolent clinical course. Fewer than 5% of patients present with advanced disease, and prognosis is more favourable compared with ccRCC [22].

### 2.4. Medullary

Medullary RCC is a rare form of RCC that is associated with poorer survival. It occurs more frequently in young African males with sickle cell trait [23]. Molecular pathogenesis is similar to urothelial carcinoma, with loss of SMARCB1, NOTCH2 amplifications and deletions of NOTCH1/NOTCH3 commonly seen [24]. Medullary RCC tends to be resistant to both cytokine and chemotherapy [23]. 

### 2.5. Collecting Duct

Collecting duct/Bellini duct RCC arises from the principal cells of renal medullary collecting ducts [25]. Histologically it is characterized by desmoplastic stroma, tubular or tubule-papillary infiltration of the renal parenchyma and high-grade nuclear features [26]. Collecting duct tumours are associated with a poor prognosis, and therefore early histological identification is imperative [27]. Next generation sequencing in a small group of patients with collecting duct RCC has revealed molecular abnormalities in *SETD2*, *CDKN2A*, *SMARCB1* and *HF2* [28].

### 2.6. Sarcomatoid and Rhabdoid Dedifferentiation

Though not categorized as subtypes of nccRCC, note should be made of the phenomena of sarcomatoid and rhabdoid dedifferentiation, which can be seen in approximately 10–15% of RCC, often co-existing with other RCC histologies (e.g., clear cell, papillary, etc.) [29]. Morphologically, sarcomatoid dedifferentiation arises from the process of epithelial-mesenchymal transition. Sarcomatoid RCC therefore contains both carcinoma and sarcomatoid-type histological features [30]. Rhabdoid dedifferentiation is characterized by clusters of large epithelioid cells with vesicular nuclei, prominent nucleoli and paranuclear intracytoplasmic hyaline inclusions [31]. Sarcomatoid and rhabdoid dedifferentiation can occur either concomitantly or independently of each other [32]. These tumours are known to be biologically aggressive and therefore confer a poor prognosis [33,34]. 

Transcriptomic analyses have demonstrated the role of *MYC* overexpression, *BAP1* mutations and *CDKN2A* deletions in the pathogenesis and aggressive course of sarcomatoid and rhabdoid dedifferentiated RCC [29]. These tumours are known to have an ‘immune-inflamed phenotype’, typified by immune activation, higher PD-L1 expression, upregulation of antigen presenting machinery genes and enhanced infiltration of cytotoxic immune cells [29]. This translates into the clinical setting, as demonstrated through the responsiveness of these tumours to IO, with response rates of 47–61% and overall survival benefit over sunitinib seen in subgroup analyses of Phase III ccRCC trials [35,36,37]. 

## 3. Systemic Therapy

A greater understanding of the molecular biology and pathogenesis of nccRCC has led to a growing number of trials exploring therapeutic agents in this context. Mirroring the trials being conducted in the ccRCC space, both immune checkpoint inhibition and targeted therapy are being investigated. Of note, local therapies such as surgery and ablative techniques (e.g., radiofrequency, microwave and cryoablation) can be utilized in the management of early stage nccRCC [38]. However, it is unclear if differences exist between outcomes in ccRCC and nccRCC managed with local therapies, as comparative studies have not been conducted. As such, systemic therapy will be the focus of this discussion.

Retrospective studies have demonstrated efficacy of IO (ORR 10–35%), and targeted therapies (ORR 15–30%) in nccRCC, although it must be recognised that sample sizes are small and study populations are heterogeneous in these analyses (Table 2).

These data do not demonstrate major differences between survival outcomes with use of targeted therapies vs. IO. Of note, poor outcomes were seen with mTOR inhibitors. Additionally, chromophobe tumours responded poorly to IO, although higher response rates were observed with combination ipilimumab/nivolumab in this subgroup. Results must be interpreted with caution, however, due to the heterogeneity of trial populations, small sample sizes and the retrospective nature of these studies. 

### 3.1. Immunotherapy

To date, other than sarcomatoid and rhabdoid dedifferentiation, no biomarkers have been able to reliably predict response to IO in renal cell carcinoma. PD-L1 expression was shown to vary between nccRCC subtype in a series that demonstrated PD-L1 positivity of 5.6% in chromophobe RCC, 10% in papillary RCC, 20% in Xp11.2 translocation RCC and 20% in collecting duct carcinoma [52]. Broadly speaking, RCC is a tumour with low tumour mutational burden (TMB), with a median of 1.1 mutations/Mb. Of RCC subtypes, lowest TMB is seen with chromophobe RCC (<1 mutation/Mb) [53]. As such, chromophobe RCC is thought to exhibit an immunologically ‘cold’ phenotype. Interestingly, however, both chromophobe and papillary RCC were shown to have the highest insertion deletion (indel) rates proportional to TMB in a pan-cancer whole-exome sequencing analysis of nineteen solid tumour types [54]. Indels are known to generate neoantigenic peptides, and therefore hypothesised to correlate with antitumour response to IO [54].

Notably, IO response rates were poorer in chromophobe RCC in comparison with other nccRCC subtypes in the majority of retrospective analyses listed in Table 2. This may add weight to the hypothesis that chromophobe tumours are indeed immunologically ‘cold’. 

Building upon retrospective data, evidence to support use of either single agent or doublet IO in this space was further substantiated in prospective single arm studies (Table 3). Parallel ccRCC cohorts in KEYNOTE-427, Checkmate-374 and HCRN GU16-260 demonstrated superior response rates of immunotherapy in ccRCC when compared with nccRCC [55,56,57]. 

### 3.2. Targeted Therapy

Prospective studies have demonstrated activity of mTOR inhibitors and anti-angiogenic VEGF tyrosine kinase inhibitors (TKIs) inhibitors against nccRCC (Table 4). Angiogenesis has been identified as a hallmark of RCC biology [60]. Activation of mTOR is known to lead to angiogenesis through HIF-1α dependent and independent factors, including modulation of nitric oxide and angiopoietins [61]. Head-to-head trials have demonstrated superior outcomes with sunitinib compared with everolimus [62,63,64], although these findings were not statistically significant and conclusions must be cautiously interpreted owing to heterogeneous trial populations, small cohorts and crossover to the sunitinib arm in the ESPN trial [63]. 

The MET proto-oncogene is implicated in the tumorigenesis of papillary RCC, particularly type 1 papillary RCC. Mechanisms of MET overexpression include gene amplification and fusions, receptor mutations, increased copy number of chromosome 7, and activating point mutations [16]. Though activity of MET inhibitors has been explored in this space, results have been disappointing in the unselected population (Table 5). The SAVOIR study is the only study to date with central confirmation of MET as a driver, demonstrating superior outcomes with the MET inhibitor savolitinib in comparison with sunitinib in an exclusively MET-driven population. Central confirmation of MET alteration is also required in the ongoing SAMETA trial (NCT05043090) [68] which aims to evaluate the efficacy of immune checkpoint and MET inhibitor as monotherapies or combination therapy in an exclusively MET-driven population. 

Knowledge of signalling pathways implicated in HLRCC such as HIF upregulation and NRF2 activation [18] has led to targeted therapy trials in this space. Response rates of approximately 50% were demonstrated with the combination of the epithelial growth factor receptor (EGFR) inhibitor erlotinib and bevacizumab, a VEGF inhibitor, in both the phase II AVATAR trial [74] and a Korean retrospective series [75]. Trials evaluating use of immune checkpoint inhibitors in this space are ongoing [18], and preclinical studies of polyadenosine diphosphate-ribose polymerase (PARP) inhibitors hold promise as a therapeutic strategy [76], although more clinical data are necessary. 

### 3.3. Combination Therapies: IO/Targeted Therapy

The interplay of tumour vessels and T-cells creates a vicious cycle within the tumour microenvironment. Tumour vessels have been shown to hinder CD8+ T cell trafficking into the tumour microenvironment, leading to impaired T-cell effector functions and T-cell killing. Additionally, VEGF has the potential to interfere with dendritic cell maturation and priming of T cells. As such, combination IO and VEGF inhibition is hypothesised to have synergistic anti-tumour activity [77]. 

Single arm prospective studies have presented efficacy data with combination IO and targeted therapy (Table 6), although more prospective, randomised trials are needed to assess superiority over other combinations and individual classes of treatment. A recent phase II single arm trial of nivolumab in combination with cabozantinib demonstrated the striking difference in response rates between patients with chromophobe (0%) vs. papillary/unclassified/translocation associated RCC (47.5%) [78]. Combination therapy with IO and MET inhibitor was explored in the CALYPSO trial in both papillary and clear cell RCC cohorts [79,80] (Table 6). In the ccRCC cohort, the primary objective of ≥50% response rate was not observed either with savolitinib monotherapy or with combination savolitinib/durvalumab. 

## 4. Future Directions

Non-clear cell RCC is a subset of genitourinary cancer that is being increasingly examined. In spite of this, however, there is still no agent or combination of agents that has parallelled response rates seen in ccRCC. As understanding of the underlying molecular pathophysiology of nccRCC grows, so does the potential for development of predictive biomarkers and future therapeutic targets. A tissue-based analysis conducted as part of the ASPEN trial (everolimus vs. sunitinib) demonstrated the relevance of particular prognostic biomarkers in nccRCC. Multivariate analysis revealed the negative and positive predictive values of p-Akt and c-kit expression respectively with regards to overall survival. Moreover, c-kit expression was predictive of benefit from everolimus, and c-MET expression correlated with a poorer response to everolimus or sunitinib [83].

The prognostic and predictive value of PD-L1 level and TMB in nccRCC is unclear, although higher PD-L1 level has been shown to correlate with poorer survival in several studies in the metastatic RCC space (inclusive of both clear cell and non-clear cell RCC) [84].Within the clear cell RCC context, recent data have highlighted the utility of metabolic profiling in predicting clinical outcomes including prognosis and response to therapies through a combination of metabolomics, lipidomics and transcriptomics. There is therefore scope for similar investigation to be prospectively conducted in the non-clear cell RCC space [85].

Other molecular targets are currently under investigation. Preclinical studies have demonstrated the oncogenicity of Hypoxia-inducible factor 1-alpha (HIF-1α) in ccRCC and FH deficient nccRCC [86]. Increased HIF-1α activity results from loss of function of the VHL tumour suppressor gene [86]. Further exploration of this target has led to development of HIF inhibitors. The role of Bezultifan, an oral HIF-2α inhibitor, in VHL associated RCC has been studied in a recent open label phase I/II study in patients, demonstrating an ORR of 49.2% [87]. Fumarate hydratase mutations—which are commonly seen in hereditary type II papillary RCC and are characterised by an aggressive clinical course—have also been hypothesised to lead to HIF-1α overexpression through intracellular accumulation of the oncometabolite fumarate, and consequent destabilisation of HIF-1α [88]. As such, the role of HIF-1α in FH-deficient RCC is ongoingly being examined. 

## 5. Discussion

Though historically poorly characterised, our understanding of nccRCC is rapidly expanding. It is imperative to recognise that nccRCC subtypes exhibit heterogeneity. Recent advances in genomic profiling have enabled identification of underlying molecular abnormalities unique to the tumorigenesis of each subtype of this disease. Prototypically, discovery of the role of MET alterations in hereditary papillary RCC has subsequently led to investigation of its role in the sporadic form of the disease, in turn resulting in therapeutic strategies with several MET inhibitor trials. In a similar vein, the pathogenic role of FH gene mutations in HLRCC has also led to exploration of HIF inhibitors as a therapeutic class. Synthesis of the aforementioned trials raises the question of the optimal first line management strategy of nccRCC subtypes. It is important to acknowledge that to date, only single arm immunotherapy trials have been conducted in this space, and no head-to-head studies of checkpoint inhibitors vs. targeted therapies exist. Indirect comparisons would suggest that response rates to checkpoint inhibitors are superior to targeted therapies with some exceptions (e.g., chromophobe RCC); however, there is no clear overall survival benefit. Additionally, trial populations are heterogeneous in subtype frequency, and inter-trial population differences exist. Given that nccRCC subtypes are biologically and phenotypically distinct, it is also important to recognise variability in efficacy of therapeutic classes between subtypes. In the majority of the listed trials, sample sizes of distinct nccRCC subtypes are small and difficult to draw firm conclusions from.

KEYNOTE-427, the largest prospective immunotherapy trial in the nccRCC space, has demonstrated a discrepancy in ORR with pembrolizumab between papillary and chromophobe RCC (28.8 vs. 9.5%, n = 21 in chromophobe subgroup) [55]. This could reflect the immunologically ‘cold’ phenotype of chromophobe RCC described in the literature [51,52]. Poor response of chromophobe RCC to IO has also been demonstrated in several retrospective analyses. In contrast, an ORR of 29% was observed with nivolumab in the chromophobe cohort of the CheckMate-374 study, albeit with a smaller sample size (n = 7) [55]. As such, it is unclear if poorer response to IO is unequivocally seen with chromophobe RCC. In light of this data, it may seem reasonable to pursue targeted therapy as a first line treatment strategy in patients with chromophobe RCC, whilst further data are awaited. The findings of the phase II single arm nivolumab/cabozantinib study demonstrating no responses in the chromophobe cohort [78] suggest that response rates are not superior with the addition of IO to targeted therapy in this subgroup.

In contrast, checkpoint inhibitors would seem to be preferable to TKI therapy in papillary and other subtypes of RCC, given suggestion of improved response rates.

MET inhibition may prove to be a viable strategy in patients with papillary RCC and confirmed MET alterations, though minimal data exist in MET-exclusive populations to date. Response rates of up to 50% have been noted in MET-driven papillary RCC [69,70], albeit in small sample sizes, and these agents certainly hold promise for the future. 

Cabozantinib has demonstrated encouraging efficacy in the treatment of collecting duct RCC, a poor prognosis RCC subtype, in the phase II BONSAI trial [67]. Given that no standard treatments exist for this disease, the observed ORR of 35% suggests that this TKI could be the agent of choice for this subpopulation.

Building upon our understanding of its molecular pathophysiology, in the HLRCC population, the combination of EGFR and VEGF inhibition (erlotinib/bevacizumab) has been shown to be effective, with response rates of up to 50% seen in the AVATAR study [74] and a separate retrospective analysis [75], highlighting this as a valid therapeutic option in this subgroup.

Moving forward, early results of HIF inhibitor trials have been promising, with the oral HIF-2 inhibitor Belzutifan inducing response rates of 49% in patients with VHL-associated RCC [87]. Further studies investigating role of HIF inhibitors in FH deficient RCC such as HLRCC are required to evaluate clinical applicability of preclinical studies using these agents.

Single arm combination IO and targeted therapy trials have been promising so far, although all four studies have demonstrated superior response rates with non-chromophobe (25–60%) than chromophobe (0–15%) cohorts [78,79,81,82]. Importantly, further head-to-head trials comparing combination therapy with both IO and targeted therapies are required to clearly answer the question of whether additional benefit is gained from a combination strategy. Comparison of doublet IO to combination IO/targeted therapy would also be clinically applicable. Moreover, breakdown of efficacy by nccRCC subtype in such studies would also guide the optimal choice of first line management.

The challenges associated with conducting further trials in a heterogeneous population with small numbers of patients with each subtype of the disease must be recognised, highlighting the importance of collaborative efforts between global institutions to establish further evidence-based guidance in the understanding and management of nccRCC. 

As our understanding of nccRCC subtypes broadens, it is also important that clinically relevant prognostic and predictive biomarkers are investigated, in order to better shape the therapeutic landscape and personalise treatment for the individual patient. In particular, the value of biomarkers such as PD-L1 expression, TMB and indel rates have not been convincingly predictive of response to immunotherapy, and more work is certainly required in this sphere. 

## 6. Conclusions

Non-clear cell RCC is comprised of a heterogeneous spectrum of biologically distinct subtypes. Understanding of the molecular pathogenesis of subtypes has significantly improved in recent times. This will hopefully pave the way for future trials investigating the role of biomarkers, and selection of the optimal therapeutic strategy in individual nccRCC subtypes. 

## Figures and Tables

**Table 1:** Incidence of RCC subtypes [12].

Subtype	% of all RCC
Papillary RCC (Types 1 and 2)	10–15
Chromophobe RCC	5
Collecting duct RCC	1
MiT family translocation RCC	1
Multilocular cystic renal neoplasm of low malignant potential	1
Medullary RCC	<1%
Tubulocystic RCC	<1%
Acquired cystic kidney disease-associated RCC	<1%
Hereditary leiomyomatosis with RCC	<1%
Succinate dehydrogenase deficient RCC	<1%
Unclassified	<1%

**Table 2:** Selected retrospective studies of targeted and immunotherapy in nccRCC [39,40,41,42,43,44,45,46,47,48,49,50,51].

Trial	Year	Treatment	Sample Size	Results			
				mOS (mths)	mPFS (mths)	ORR	Other Endpoints
Colomba et al. [39]	2017	VEGF/mTORi VEGFi: 82% mTORi: 18%	*n* = 61	VEGFi: 22.9 (95% CI 17.8–49.2) mTORi: 3.2 (95% CI 2.3–NE)	N/A	VEGFi: 28.9% mTORi: 0%	mTTF VEGFi: 8.0mths (95% CI 4.1–13.6) mTORi: 2.3mths (95% CI 0.7–8.0)
Buti et al. [40]	2017	Pazopanib	*n* = 37 Papillary: 51% Chromophobe: 24% Unclassified: 21%	17.3 (95% CI 11.5–23.0)	15.9 (95% CI 5.9–25.8)	Overall: 27% Papillary: 21% Chromophobe: 44%	DCR: 81%
Campbell et al. [41]	2018	Cabozantinib	*n* = 30	25.4 (95% CI 15.5–35.4)	8.6 (95% CI 6.1–14.7)	14.3%	DCR: 78.6%
Agarwala et al. [42]	2018	VEGF/mTORi Sorafenib: 35% Sunitinib: 22.5% Pazopanib: 20% Everolimus: 17.5%	*n* = 40 Papillary: 62.5% Sarcomatoid: 15% Chromophobe: 12.5%	11.7	N/A	N/A	mEFS: 6.1mths
Chanza et al. [43]	2019	Cabozantinib	*n* = 112 Papillary: 59% Translocation: 15% Unclassified: 13% Chromophobe: 9%	12.0 (95% CI 9.2–17.0)	7.0 (95% CI 5.7–9.0)	Overall: 27% Papillary: 27% Chromophobe: 30% Collecting duct: 50% Unclassified: 13%	mTTF: 6.7mths
Kim et al. [44]	2019	VEGFi vs. mTORi	*n* = 156 Papillary: 59.6% Chromophobe: 12.8% Collecting duct: 11.5% Unclassified: 10.3%	N/A	10.0 vs. 5.0 (*p* = 0.0275)	N/A	mCSS: 27.0 vs. 16.0 (*p* = 0.1706)
Choudhary et al. [45]	2020	VEGFi Sorafenib: 39.2% Sunitinib: 27.4% Pazopanib: 21.6%	*n* = 139 Papillary: 76.2% Chromophobe: 7.9% Sarcomatoid: 6.5% Unclassified: 1.4%	11.9 (95% CI 5.4–18.4)	6.0 (95% CI 2.4-9.6)	16.6%	DCR: 54.1%
McKay et al. [46]	2018	PD-1/PD-L1 inhibitors	*n* = 43 Papillary: 33% Chromophobe: 23% Unclassified: 21% Sarcomatoid: 16% Translocation: 7%	12.9 (95% CI 7.4–NR)	N/A	Overall: 19% Papillary: 29% Chromophobe: 0% Translocation: 33% Unclassified: 0%	mTTF: 4.0mths (95% CI 2.8–5.5)
Gupta et al. [47]	2020	Ipilimumab/ Nivolumab	*n* = 18	N/A	7.1	Overall: 33.3% Chromophobe: 20% Medullary: 0% Papillary T2: 50% Translocation: 0% Unclassified: 33.3%	N/A
Chahoud et al. [48]	2020	Nivolumab	*n* = 40 Papillary: 30% Unclassified: 27.5% Chromophobe: 12.5% Translocation: 7.5%	21.7 (95% CI 7.83–NR)	4.9 (95% CI 3.53–10.27)	Overall: 21.6% Papillary T1: 25% Papillary T2: 0% Chromophobe: 0% Translocation: 0% Unclassified: 44.4%	DCR: 53.4%
Koshkin et al. [49]	2018	Nivolumab	*n* = 41 Papillary: 39% Unclassified: 34% Chromophobe: 12% Collecting duct: 10%	NR	3.5 (95% CI 1.9–5.0)	Overall: 20% Papillary: 14% Chromophobe: 0% Collecting duct: 25% Translocation: 0% Unclassified: 36%	10mth OS: 68%
Schwartzmann et al. [50]	2020	Nivolumab: 46% Ipilimumab/ nivolumab: 54%	*n* = 28 Unclassified: 42.9% Papillary: 28.6% Chromophobe: 10.7% HLRCC: 10.7%	15.9 (95% CI 5.9–25.9)	N/A	10.7%	mTNT: 4.9 (95% CI 1.7–8.1)
ORACLE [51]	2021	IO/VEGF (Pembrolizumab/ axitinib, atezolizumab/ bevacizumab, avelumab/ axitinib)	*n* = 19	24.7	16.8	21%	mDOR: 23.6mths DCR: 69%
		IO/IO (ipilimumab/ nivolumab)	*n* = 40	19.2	13.6	19%	mDOR: 13.6mths DCR: 46%
		VEGF/mTORi (lenvatinib/ everolimus)	*n* = 7	23.1	NR	0%	mDOR: NR DCR: 72%

mOS: median overall survival, mPFS: median progression free survival, ORR: objective response rate, mEFS: median event free survival, DCR: disease control rate, mTTF: median time to treatment failure, mCSS: median cancer specific survival, mTNT: median time to next treatment, mDOR: median duration of response.

**Table 3:** Prospective single arm immunotherapy trials in nccRCC [55,56,57,58,59].

Trial	Phase	Cohort	Treatment	Sample Size	Efficacy		
					mOS (mths)	mPFS (mths)	ORR
KEYNOTE-427 [55]	II	A (Clear Cell)	Pembrolizumab	*n* = 110	NR	7.1 (95% CI 5.6–11.0)	36.4%
		B (Non-clear cell)		*n* = 165 Papillary: 71.5% Unclassified: 15.8% Chromophobe: 12.7%	28.9 (95% CI 24.3-NR)	4.2 (95% CI 2.9–5.6)	26.7% Papillary: 28.8% Chromophobe: 9.5% Unclassified: 30.8%
CheckMate-374 [56]	III/IV	A (Clear Cell)	Nivolumab	*n* = 97	21.8 (95% CI 17.4-NE)	3.6 (95% CI 2.0–5.5)	22.7%
		B (Non-clear cell)		*n* = 44 Papillary: 54.5% Chromophobe: 15.9% Unclassified: 18.2% Translocation: 5% Other: 6%	16.3 (95% CI 9.2-NE)	2.2 (95% CI 1.8–5.4)	13.6%
CheckMate-920 [58]	III/IV	Cohort 2 (nccRCC with KPS ≥70%) ^	Ipilimumab/ nivolumab > nivolumab	*n* = 52 Unclassified: 42.3% Papillary: 34.6% Chromophobe: 13.5% Translocation: 3.8% Collecting duct: 3.8% Medullary: 1.9%	21.2 (95% CI 16.6-NE)	3.7 (95% CI 2.7–4.6)	19.6%
HCRN GU16-260 [57]	II	A (Clear Cell)	Nivolumab (Part A) +/− ipilimumab * (Part B)	*n* = 123	N/A	7.4 (95% CI 5.5–10.9)	Part A: 29.3% Part B: 11%
		B (Non-clear cell)		*n* = 35 Papillary: 54% Chromophobe: 17% Unclassified: 29% Part B: *n* = 16	N/A	4.0 (95% CI 2.7–4.3)	Part A: 14.3% Part B: 6%
UNISoN (ANZUP 1602) [59]	II	N/A (nccRCC cohort only)	Nivolumab +/− ipilimumab **	*n* = 85	Part 1+2: 24 (95% CI 16–28) Part 2 (nivo+ipi): 10 (95% CI 6–17)	Part 2: 2.6 (95% CI 2.2–3.8)	Nivo: 17% Nivo + ipi: 10%

KPS: Karnosfky Performance Score. ^CheckMate-920 included 4 cohorts: predominantly ccRCC with KPS ≥ 70% (cohort 1), nccRCC with KPS≥70% (cohort 2), cc/nccRCC with nonactive brain metastases and KPS≥70% (cohort 3), cc/nccRCC with KPS 50–60% (cohort 4). * Salvage ipilimumab (1mg/kg) and nivolumab (3mg/kg) was administered if PD or SD was found 48 weeks after initiation. ** Salvage ipilimumab (1 mg/kg) was administered in patients refractory to single agent nivolumab at 3 months.

**Table 4:** Prospective targeted therapy trials in nccRCC [62,63,64,65,66,67].

Trial	Phase	Treatment	Comparator	Sample Size	Efficacy		
					mOS (mths)	mPFS (mths)	ORR
ASPEN [62]	II	Sunitinib	Everolimus	*n* = 108	31.5 vs. 13.2 HR 1.12 (95% CI 0.7–2.1, *p* = 0.60)	8.3 vs. 5.6 HR 1.41 (80% CI 1.03–1.92, *p* = 0.16)	18% vs. 9%
ESPN [63]	II	Sunitinib	Everolimus	*n* = 68 (Crossover allowed at PD)	16.2 vs. 14.9 (*p* = 0.18)	1L: 6.1 vs. 4.1 (*p* = 0.6) 2L: 1.8 vs. 2.8 (*p* = 0.6)	1L: 9% vs. 3% 2L: 9.5% vs. 8.6%
RECORD-3 [64]	II	1L everolimus/ 2L sunitinib	1L sunitinib/ 2L everolimus	*n* = 471	22.4 (95% CI 18.6-33.3) vs 29.5 (95% CI 22.8–33.1)	21.7 (95% CI 15.1–26.7) vs 22.2 (95% CI 16.0–29.8)	N/A
RAPTOR [65]	II	Everolimus	N/A	*n* = 92 Papillary: 78%	21.4 (95% CI 15.4–28.4)	T1 Papillary: 7.9 (95% CI 2.1–11.0) T2 Papillary: 5.1 (95% CI 3.3–5.5)	1%
ARCC [66]	III	Interferon alfa	Temsirolimus/Combination therapy	*n* = 626	7.3 vs. 10.9 vs. 8.4	3.1 vs. 5.5 vs. 4.7	4.8% vs. 8.6% vs. 8.1%
BONSAI [67]	II	Cabozantinib	N/A	*n* = 23 (Collecting duct RCC only)	7.0 (95% CI 3–31)	4.0 (95% CI 3–13)	35% (95% CI 15–57)

**Table 5:** MET inhibitor trials in papillary RCC [69,70,71,72,73].

Trial	Phase	Treatment	Sample Size	Efficacy		
				mOS (mths)	mPFS (mths)	ORR
CREATE (T1 Papillary RCC) [69]	II	Crizotinib	*n* = 23 (MET-altered: n = 4)	N/A	N/A	MET positive cohort: 50% (2/4 patients) MET negative cohort: 6.3% (1/16 patients)
NCT00726323 [70]	II	Foretinib	*n* = 74 (MET-altered: n = 36)	NR	9.3	Overall population: 13.5% Germline MET mutation positive: 50% (5/10 patients) Germline MET mutation negative: 9% (5/57 patients)
SWOG S1107 [71]	II	Tivantinib/erlotinib vs Tivantinib	*n* = 50 Tivantinib/ erlotinib: *n* = 25 Tivantinib: *n* = 25 (MET-altered: n = 1)	10.3 vs. 11.3	5.4 vs. 2.0	0% in both arms
SAVOIR [72]	III	Savolitinib vs. Sunitinib	*n* = 60 (all MET-driven) Savolitinib: *n* = 33 Sunitinib: *n* = 27	NR vs. 13.2 (HR 0.51, 95% CI 0.2–1.2; *p* = 0.11)	7.0 vs. 5.6 (HR 0.71; 95% CI 0.4–1.4)	27% vs. 7%
PAPMET [73]	II	Savolitinib/ Crizotinib/ Sunitinib/ Cabozantinib	*n* = 152 (MET testing not performed)	N/A	No improvement in PFS with savolitinib/ crizotinib vs. VEGFi (halted early after prespecified futility analysis)	N/A
		Cabozantinib vs. Sunitinib		N/A	9.0 vs. 5.6 HR 0.60	

**Table 6:** Completed single arm studies of IO plus targeted therapy in nccRCC [78,79,81,82].

Trial	Phase	Treatment	Sample Size	Efficacy
NCT02724878 [81]	II	Atezolizumab/ Bevacizumab	*n* = 60 Papillary: *n* = 12 Chromophobe: *n* = 10 Unclassified: *n* = 9 Collecting duct: *n* = 5 Medullary: *n* = 1	ORR: 33% Papillary: 25% Chromophobe: 10% Unclassified: 33% Collecting duct: 40% Medullary: 100%
COSMIC-021 [82]	Ib/II	Atezolizumab/ Cabozantinib	*n* = 32 (nccRCC cohort) Papillary: *n* = 15 Chromophobe: *n* = 9 Other: *n* = 7	mPFS: 9.5mths ORR 31% (80% CI: 20-44) Papillary: 40% Chromophobe: 14% Other: 60%
CALYPSO [79]	I/II	Durvalumab/ Savolitinib	*n* = 41 Papillary: *n* = 40	ORR 27% mOS: 12.3mths mPFS: 4.9mths
NCT03635892 [78]	II	Nivolumab/ Cabozantinib	*n* = 47 Cohort 1 (papillary, unclassified, translocation associated RCC): *n* = 40 Cohort 2 (chromophobe): *n* = 7	Cohort 1: ORR 47.5%, mPFS 12.5mths, mOS: 28mths Cohort 2: ORR 0%
